# Impact of the 2009 H1N1 Pandemic on Age-Specific Epidemic Curves of Other Respiratory Viruses: A Comparison of Pre-Pandemic, Pandemic and Post-Pandemic Periods in a Subtropical City

**DOI:** 10.1371/journal.pone.0125447

**Published:** 2015-04-30

**Authors:** Lin Yang, Kwok Hung Chan, Lorna K. P. Suen, King Pan Chan, Xiling Wang, Peihua Cao, Daihai He, J. S. Malik Peiris, Chit Ming Wong

**Affiliations:** 1 School of Nursing, The Hong Kong Polytechnic University, Hong Kong; 2 Department of Microbiology, The University of Hong Kong, Hong Kong; 3 Division of Epidemiology and Biostatistics, School of Public Health, The University of Hong Kong, Hong Kong; 4 Department of Applied Mathematics, The Hong Kong Polytechnic University, Hong Kong; 5 State Key Laboratory of Emerging Infectious Disease, School of Public Health, The University of Hong Kong, Hong Kong; Harvard School of Public Health, UNITED STATES

## Abstract

**Background:**

The 2009 H1N1 influenza pandemic caused offseason peaks in temperate regions but coincided with the summer epidemic of seasonal influenza and other common respiratory viruses in subtropical Hong Kong. This study was aimed to investigate the impact of the pandemic on age-specific epidemic curves of other respiratory viruses.

**Methods:**

Weekly laboratory-confirmed cases of influenza A (subtypes seasonal A(H1N1), A(H3N2), pandemic virus A(H1N1)pdm09), influenza B, respiratory syncytial virus (RSV), adenovirus and parainfluenza were obtained from 2004 to 2013. Age-specific epidemic curves of viruses other than A(H1N1)pdm09 were compared between the pre-pandemic (May 2004 – April 2009), pandemic (May 2009 – April 2010) and post-pandemic periods (May 2010 – April 2013).

**Results:**

There were two peaks of A(H1N1)pdm09 in Hong Kong, the first in September 2009 and the second in February 2011. The infection rate was found highest in young children in both waves, but markedly fewer cases in school children were recorded in the second wave than in the first wave. Positive proportions of viruses other than A(H1N1)pdm09 markedly decreased in all age groups during the first pandemic wave. After the first wave of the pandemic, the positive proportion of A(H3N2) increased, but those of B and RSV remained slightly lower than their pre-pandemic proportions. Changes in seasonal pattern and epidemic peak time were also observed, but inconsistent across virus-age groups.

**Conclusion:**

Our findings provide some evidence that age distribution, seasonal pattern and peak time of other respiratory viruses have changed since the pandemic. These changes could be the result of immune interference and changing health seeking behavior, but the mechanism behind still needs further investigations.

## Introduction

Previous studies have proposed a hypothesis of viral interference between influenza and other respiratory viruses [1]. Experiments have shown that respiratory virus infection can stimulate temporary non-specific innate immunity, thereby protecting the host from secondary infections of other viruses [2]. This finding is further supported by the observation that children who receive trivalent influenza inactivated vaccines have an increased risk of infection of non-influenza viruses [3]. However, other studies have failed to find an association between influenza vaccination and non-influenza infections [4]. Investigations into the age-specific epidemic curves of multiple respiratory viruses across different regions and climates can help resolve the controversy over the potential interference between different viruses. The 2009 H1N1 pandemic was characterized with an offseason surge of infected cases in temperate regions, with an age distribution shift towards children and young adults, which is distinct from seasonal influenza outbreaks [5]. A similar age shift was also observed in the subtropical city of Hong Kong, but the pandemic coincided with the summer epidemic of seasonal influenza. We hypothesize that the emergence of this new influenza virus strain could have interrupted the regular circulation of other respiratory viruses through the viral interference of competing for entry sites and changing the preexisting innate immunity. This viral interference could be reflected by the change of age distribution and seasonal variations of the respiratory viruses other than influenza, such as late (or early) peaks and altered seasonal patterns during and after the pandemic. In this study, we utilized 10 years of age-specific surveillance data on common respiratory viruses of influenza, RSV, adenovirus and parainfluenza in subtropical Hong Kong, with the aim to assess the impact of the 2009 H1N1 pandemic on the age-specific epidemic curves of other respiratory viruses by comparing the periods before, during, and after the pandemic.

## Methods

We analyzed the age profile of 120571 cases with influenza like symptoms and specimens tested the microbiology laboratory of Queen Mary Hospital (QMH) during the study period of January 2004 to December 2013. This laboratory is one of largest sentinel surveillance laboratories in Hong Kong and covers 20% of the population. During the whole study period, direct immunofluorescence (IF) tests were conducted to test influenza (type A or B), RSV, adenovirus and parainfluenza (type 1,2,3) [6]. Influenza A positive specimens were subtyped into seasonal A(H3N2) and A(H1N1) by viral culture and also subtyped into the pandemic virus A(H1N1)pdm09 by RT-PCR during the 2009 H1N1 pandemic until early 2010. Non-subtyped influenza A positive specimens were allocated into three subtypes by annual ratio of total numbers of each subtype. Because the A(H1N1)pdm09 did not emerge in Hong Kong until May 2009, the annual ratio of year 2009 was separately calculated for the first 17 weeks of 2009 and the remaining 35 weeks. Weekly age-specific numbers of positive specimens were aggregated into the age groups of 0–4y, 5–17y, 18–64y, 65y and above. Weekly age-specific positive proportions were calculated as weekly age-specific numbers of positive specimens divided by weekly total numbers of specimens tested for each age group.

To assess the impact of the 2009 pandemic on virus circulation, the overall positive proportions in five pre-pandemic seasons (2 May 2004 to 25 April 2009), one pandemic season (26 April 2009 to 25 April 2010), and three post-pandemic seasons (26 April 2010 to 20 April 2013) were compared by Chi-square tests. Here influenza season was defined as week 18 of the present year to week 17 of the following year to ensure that other seasonal factors were comparable between the periods. Hence the length of these nine seasons was shorter than the entire study period of January 2004 to December 2013. Positive proportion was used instead of positive number because the former was less affected by yearly variations in the number of specimens collected for each age group.

We used wavelet analysis [7, 8] to assess the seasonal patterns of age-specific virus epidemic curves. Unlike other approaches that decompose the temporal variations of time series, wavelet analysis does not require stationary datasets and thus is suitable for virological data in Hong Kong given their unpredictable seasonality. This approach has been adopted to explore the spatiotemporal patterns of influenza activity across different states of the United States [9] as well as the synchrony of different surveillance data in Hong Kong [10]. A detailed description of wavelet analysis can be found in references [8, 11]. Briefly, Morlet wavelets at the frequency ranging from biannual to quarterly cycles were fitted to age-specific virology data to reveal the temporal change in relative dominance of these cycles. Similar to R^2^ in linear models, wavelet power spectrum was calculated at each time point for each frequency to quantify the relative dominance of these cycles over time. Wavelet power spectrum ranges from 0 to 1, with the highest indicating the complete dominance and the lowest the complete missing of certain cycle.

We calculated the mean week of the epidemics (MWE) to compare the epidemic peak time in the pre- and post-pandemic seasons [12]:

$${\text{MWE}}_{i}=\sum v_{i}{}_{t}*t/\sum v_{i}{}_{\text{t}}$$

where *v_i_* denotes the number of laboratory confirmed cases at week *t* (= 1,2,…,49, -2, -1,0) of season *i*. Because of two-peak patterns shown in most respiratory viruses, MWE was calculated separately for the warm (weeks 18 to 49) and cool season (week 50 to week 17 of the following year) within each influenza season. Here we replaced the week numbers 50 to 52 by -2 to 0 to avoid the dominance of these large numbers. Some virus-age groups were excluded from this analysis, due to small numbers of cases (annual total < 20 in half of study years). All analyses were conducted in R package version 2.12.2 (R Foundation for Statistical Computing, Vienna, Austria). Wavelet analysis was conducted by using the sowas and Rwave packages of R [7, 13]. Ethical approval was obtained from the Institutional Review Board of the University of Hong Kong/Hospital Authority Hong Kong West Cluster (UV11-264). Written consent was waived as only the aggregated data were used in analysis.

## Results

A total of 120 571 specimens were collected during the entire study period of January 2004 to December 2013. We examined the age profile of 9 952, 1 509, 5 012, 1 467, and 3 391 cases with laboratory-confirmed infections of influenza A, influenza B, RSV, adenovirus, and parainfluenza, respectively. During the pandemic period of 26 April 2009 to 24 April 2010, the positive rate of A(H1N1)pdm09 was high among school children and adults but low in the elderly. After the pandemic, the positive rate of A(H1N1)pdm09 became low in school children and adults. Compared with those in the pre-pandemic period, a markedly decrease of positive proportions in the pandemic was observed for nearly all the non-pandemic viruses; RSV and A(H3N2) slightly increased in the 0–4y age group, but only RSV reached statistical significance (Table 1). After the pandemic, seasonal A(H1N1) almost completely disappeared from the Hong Kong population, and positive proportions of A(H3N2) were higher than those in the pre-pandemic, but for influenza B the proportions were slightly lower in all age groups.

**Table 1:** Annual age-specific average numbers (No), positive rates per 100 000 person-years (Rate) and positive proportions (Prop) during the pre-pandemic, pandemic and post-pandemic periods.

Virus	Age	Pre-pandemic^a^			Pandemic^b^			Post-pandemic^c^			*p* value ^d^		
		No	Rate	Prop	No	Rate	Prop	No	Rate	Prop	Pre- vs. pandemic	Post- vs. pandemic	Pre- vs. Post-pandemic
A(H1N1)	0-4y	278	120.5	1.5	20	43.4	0.5	6	13.0	0.1	<0.001	<0.001	<0.001
	5-17y	239	33.6	3.7	3	2.1	0.1	1	0.2	0.0	<0.001	0.398	<0.001
	18-64y	170	3.8	2.1	33	3.7	0.5	8	0.9	0.1	<0.001	<0.001	<0.001
	65y+	110	12.1	0.7	31	17.1	0.3	1	0.2	0.0	<0.001	<0.001	<0.001
A(H3N2)	0-4y	625	270.9	3.5	159	344.6	3.9	439	951.6	5.2	0.179	0.002	<0.001
	5-17y	250	35.1	3.9	60	42.2	2.7	165	38.7	5.3	0.012	<0.001	0.002
	18-64y	345	7.6	4.3	243	26.9	3.8	324	35.9	4.3	0.131	0.092	0.859
	65y+	806	88.7	5.5	276	151.9	3.1	1255	230.3	6.1	<0.001	<0.001	0.018
A(H1N1)pdm09	0-4y	NA	NA	NA	443	960.2	10.9	298	645.9	3.5	NA	<0.001	NA
	5-17y	NA	NA	NA	987	693.7	44.3	116	27.2	3.7	NA	<0.001	NA
	18-64y	NA	NA	NA	938	103.9	14.5	252	27.9	3.4	NA	<0.001	NA
	65y+	NA	NA	NA	97	53.4	1.1	121	22.2	0.6	NA	<0.001	NA
Influenza B	0-4y	251	108.8	1.4	23	49.9	0.6	91	197.2	1.1	<0.001	0.007	0.039
	5-17y	340	47.8	5.3	50	35.1	2.2	124	29.0	4.0	<0.001	0.001	0.007
	18-64y	125	2.8	1.5	31	3.4	0.5	105	11.6	1.4	<0.001	<0.001	0.511
	65y+	130	14.3	0.9	15	8.3	0.2	165	30.3	0.8	<0.001	<0.001	0.425
RSV	0-4y	1827	792.0	10.1	469	1016.6	11.5	795	1723.2	9.4	0.009	<0.001	0.066
	5-17y	90	12.7	1.4	22	15.5	1.0	52	12.2	1.7	0.177	0.048	0.340
	18-64y	148	3.3	1.8	75	8.3	1.2	90	10.0	1.2	0.001	0.867	0.002
	65y+	376	41.4	2.6	182	100.2	2.0	350	64.2	1.7	0.012	0.048	<0.001
Adenovirus	0-4y	625	270.9	3.5	57	123.6	1.4	245	531.1	2.9	<0.001	<0.001	0.017
	5-17y	257	36.1	4.0	10	7.0	0.4	110	25.8	3.5	<0.001	<0.001	0.310
	18-64y	20	0.4	0.2	5	0.6	0.1	10	1.1	0.1	0.024	0.449	0.153
	65y+	4	0.4	0.0	1	0.6	0.0	2	0.4	0.0	0.719	1.000	0.406
Parainfluenza	0-4y	1135	492.0	6.3	198	429.2	4.9	508	1101.1	6.0	0.001	0.011	0.380
	5-17y	96	13.5	1.5	32	22.5	1.4	57	13.4	1.8	0.951	0.321	0.243
	18-64y	116	2.6	1.4	62	6.9	1.0	96	10.6	1.3	0.012	0.083	0.463
	65y+	296	32.6	2.0	115	63.3	1.3	315	57.8	1.5	<0.001	0.126	0.001

RSV, respiratory syncytial virus; NA, not available; NS, not significant

*** *p* <0.001,

** *p* <0.01,

* *p* < 0.05

^a^ Pre-pandemic period is 2 May 2004–25 April 2009.

^b ^Pandemic period is 26 April 2009–24 April 2010.

^c^ Post-pandemic period is 26 April 2010 to 20 April 2013.

^*d*^ *p*-value of Chi-square tests for positive proportions of the pandemic (or post-pandemic) and pre-pandemic periods. Fisher exact test was used instead when cell counts were lower than five.

**Fig 1:**
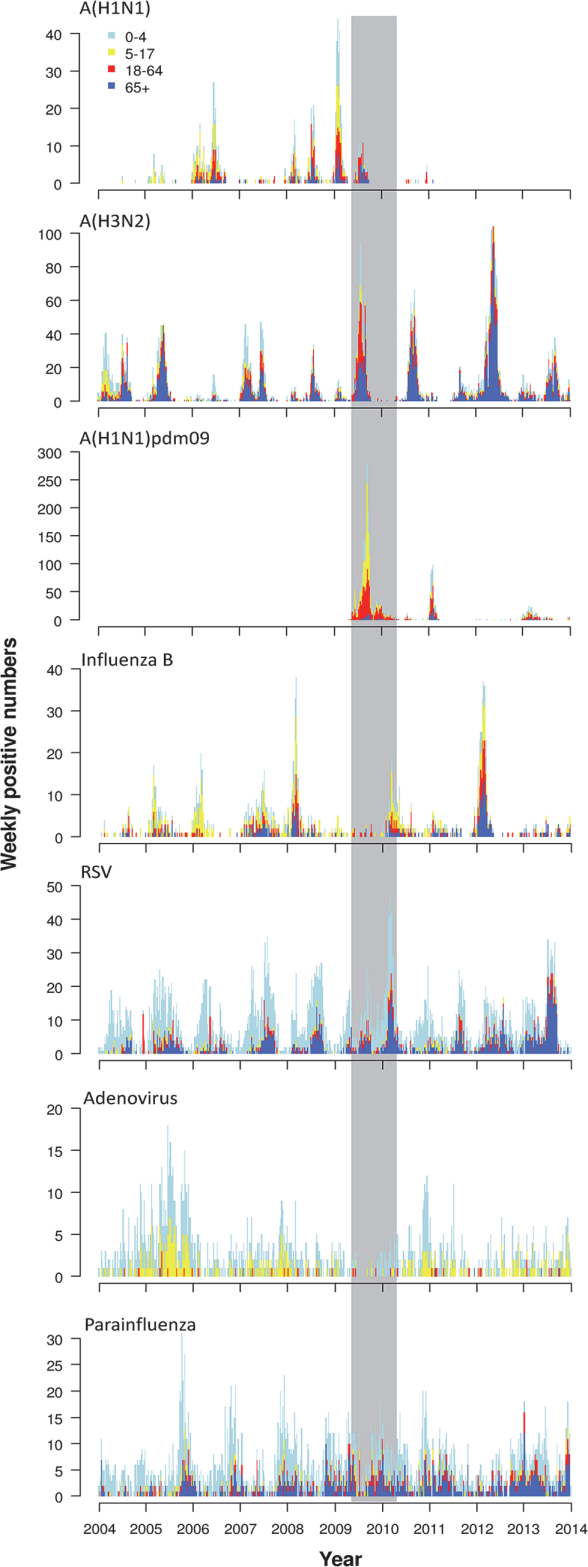
Weekly numbers of laboratory confirmed cases with influenza A (subtypes A(H1N1), A(H3N2) and A(H1N1)pdm09), influenza B, respiratory syncytial virus (RSV), adenovirus or parainfluenza by age group, Hong Kong, 2004–2010. The pandemic period of 26 April 2009–24 April 2010 is highlighted in gray band.

Age-specific epidemic curves of influenza A(H1N1), A(H3N2), A(H1N1)pdm09, influenza B, RSV, adenovirus and parainfluenza are shown in Fig 1. Seasonal subtype A(H1N1) infected many children aged below 18 years in all the study years but only widely circulated in some years (2006, 2008, and 2009). A(H3N2) was frequently isolated from all age groups except 2006, with high numbers observed in the age groups of 0–4y and 65y above. Both A(H1N1) and A(H3N2) usually showed a two-peak annual seasonal pattern, and one broad peak was occasionally observed during the study period. Influenza B had a single broad annual peak in most years but remained at low level in 2004, 2009, 2011 and 2013. The first wave of A(H1N1)pdm09 occurred around September 2009, and the second wave around February 2011. We also found that school children were more exempted from the second wave, while in the elderly more infections occurred in the second wave as compared to the first wave. RSV was characterized with broad peaks spanning over one or two months (Fig 1). Two peaks of RSV cases were observed among young children each year, but no clear patterns were observed in adults due to the limited number of cases.

**Fig 2:**
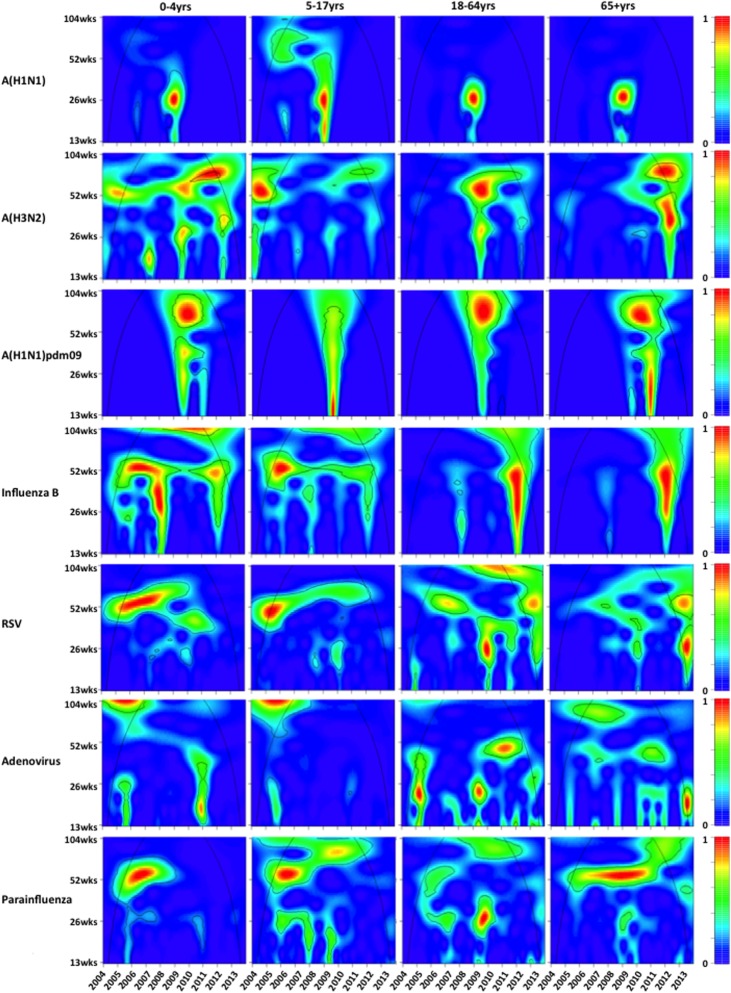
Wavelet spectrum of age- and virus-specific positive numbers. The black contour lines show the regions of power significant at the 5% level computed based on 1,000 Monte Carlo simulations. The cone of influence (black curve) indicated the region without edge effects. The power values were coded from dark blue for low power to dark red for high power, as shown in the right panel.

**Fig 3:**
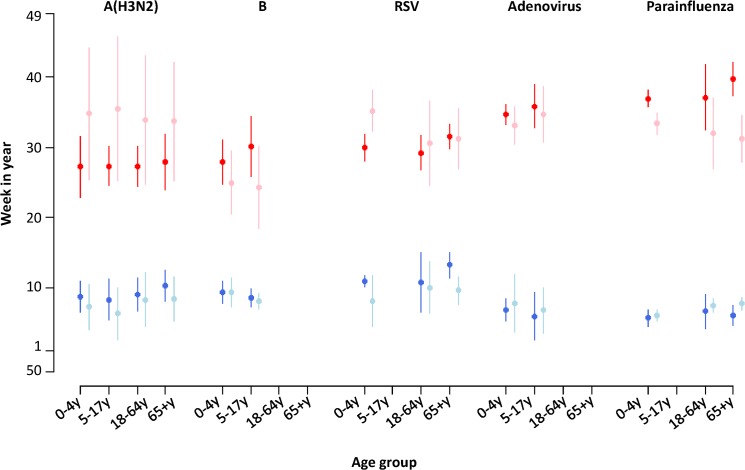
Comparison of the mean week of the epidemics (MWE) during the pre- and post-pandemic periods, for influenza A(H3N2), B, respiratory syncytial virus (RSV), adenovirus and parainfluenza. Red and blue dots represent MWE in warm season (week 19 to week 50) and cool season (week 51 to week 18 of next year) of the pre-pandemic period. Pink and light blue dots represent MWE in warm and cool season of the post-pandemic period. The vertical bars indicate ± one standard error.

All the viruses demonstrated annual and semiannual seasonal patterns across age groups (Fig 2). Annual cycle was dominant throughout the study period for A(H3N2) and B, but semiannual cycle became dominant for A(H1N1) in early 2009 before the pandemic outbreak. In contrast to seasonal influenza viruses, A(H1N1)pdm09 showed a biannual seasonal pattern, corresponding to the two waves in 2009 and 2011. RSV and parainfluenza showed a gradual shift from annual to semiannual cycle, especially in young children. The seasonal pattern of adenovirus was least clear among all the viruses. Compared to the pre-pandemic period, the MWE of A(H3N2) in all age groups dramatically increased during the post-pandemic period for the winter epidemics, but earlier peaks were observed for the summer epidemics (Fig 3). Similar changes were also observed in RSV, with the only exception of the 65y above group. Influenza B, adenovirus and parainfluenza showed slightly earlier summer peaks but delayed winter peaks in most age groups during the post-pandemic period.

## Discussion

In line with other studies in Hong Kong and other countries, we found that the first wave of the 2009 H1N1 pandemic was characterized by a shift towards younger age in infected and hospitalized cases [14, 15]. Over 95% of the pandemic cases occurred in people younger than 65 years whereas there were a much lower proportion of seasonal influenza cases in these age groups (Table 1). We also found that school children were relatively exempted from the second wave, and more infections occurred in the elderly compared to the first wave. The age shift back to older people was also observed in elsewhere [16, 17]. This age shift is likely due to the age heterogeneity in preexisting immunity obtained from the first wave. Serological studies in Hong Kong have found that 43.4% of school children aged 5–14y but only 0.77% of persons aged 60 years and over were sero-positive for A(H1N1)pdm09 after the first wave [5, 18].

Infections of one respiratory virus may stimulate temporary nonspecific immune response in human hosts and thus prevent secondary infections of other respiratory viruses, although how long such nonspecific immunity can last remains unclear [1]. For example, studies have demonstrated that children who received influenza vaccination had a significantly high infection rate of other respiratory viruses [3, 19]. By analyzing the age specific virology data of 10 years, we are able to assess the impact of the pandemic on age patterns of other respiratory viruses. We found significantly low positive proportions of non-pandemic respiratory viruses during the 2009 pandemic; the only exception was a small increase in RSV among children aged below five. Viral interference between the novel 2009 pandemic virus and other respiratory viruses, including rhinovirus, adenovirus and RSV, has also been reported in previous studies [20–22]. We also found high positive proportions of A(H3N2) in the post-pandemic periods for all age groups (except the 18–64y group), low proportions of influenza B in children, and low proportions of RSV in adults and old people, as compared with those in the pre-pandemic period. Further investigations are warranted to demonstrate whether such changes were caused by variations in virus virulence or underlying immunity.

Besides viral interference, there are many other factors that could have also played a role in regulating seasonal patterns of respiratory viruses, including health seeking behavior, preexisting immunity and vaccination history. Compared to the pre-pandemic period, we did not observe any obvious change in seasonal patterns of other respiratory viruses in the post-pandemic period, except a trend of emerging semiannual cycles in young children. Interestingly, we found earlier epidemic peaks in influenza B, adenovirus and parainfluenza after the first wave of the pandemic, but delayed peaks in A(H3N2) and RSV. There are no good explanations for such a discrepancy, but it is of note that the former group of viruses tends to cause relatively mild infections than the latter group, especially in small children [23]. We postulate that earlier peaks in mild infections could be due to changing health seeking behavior after the pandemic. Although no studies have reported the change of health seeking behavior after the pandemic in Hong Kong, obviously increased awareness to respiratory infections has been observed in Hong Kong parents after the SARS outbreak in 2003 [24]. Similarly, the increased awareness against respiratory virus infections could have resulted in instant consultations after the onset of respiratory symptoms. This change could be more evident in young children, because they have been widely reported as the high-risk group of the pandemic. Moreover the Hong Kong parents tend to be very protective and vigilant. This could be revealed by the dramatically high admission rates of acute respiratory diseases associated with influenza in Hong Kong children, as compared to those in the other regions [25]. Unfortunately, we do not have any data on health seeking behavior of the Hong Kong general population before and after the pandemic. However, it is difficult to assess the preexisting immune status for all the subjects, given the frequently occurred antigenic drifts in influenza viruses [26]. The overall vaccination rate remained extremely low in Hong Kong after the pandemic. A survey showed that only 18% of the Hong Kong population were vaccinated in the 2012/13 season, and the coverage was 28.4%, 11.0%, 8.5% and 39.1% in the age groups of 6m-5y, 6-49y, 50-64y and 65y above [27]. These rates are far below the target of 70% coverage in the Healthy People 2020 proposed by the Department of Health and Human Services of the United States [28].

Our study has several limitations. First, we assessed the impact of the pandemic on virus activities of seasonal influenza, RSV, adenovirus and parainfluenza in this study, but there are many other respiratory viruses, such as rhinovirus, enterovirus and coronavirus, that were not assessed due to the lack of surveillance data. Second, our data only cover five influenza seasons in the pre-pandemic period and three in the post-pandemic, which might not be long enough to reliably reveal the peaks of seasonal epidemics for each age group. It is possible that some of changes we observed could be due to the year-to-year variations of virus activities. Third, the sensitivity and specificity of laboratory tests may vary across age, which might have resulted in different extents of underreporting. Nevertheless, our data were collected by a single laboratory, which consistently followed the same protocol during the whole study period. Therefore this underreporting unlikely affects our comparison before and after the pandemic.

This study for the first time comprehensively analyzed a large amount of age-specific virology data in the subtropical regions, to our best knowledge. We found that the age distribution, seasonal pattern and peak time of six respiratory viruses other than A(H1N1)pdm09 have changed since the pandemic, despite that the magnitude of most changes is not significant and the patterns appear inconsistent. These changes could be the result of viral interference, or could have also been caused by changing health-seeking behavior and other unknown mechanisms. Similar studies in other regions when age specific virology data become available in a wide range could help elucidate the mechanism.

## References

[1] Cowling BJ, Nishiura H. Virus interference and estimates of influenza vaccine effectiveness from test-negative studies. Epidemiology. 2012;23:930–1. 10.1097/EDE.0b013e31826b300e 23038121

[2] McGill J, Heusel JW, Legge KL. Innate immune control and regulation of influenza virus infections. J Leukoc Biol. 2009;86:803–12. 10.1189/jlb.0509368 19643736 PMC2752015

[3] Cowling BJ, Fang VJ, Nishiura H, Chan KH, Ng S, Ip DKM, et al Increased risk of noninfluenza respiratory virus infections ssociated with receipt of inactivated influenza vaccine. Clin Infect Dis. 2012;54:1778–83. 10.1093/cid/cis307 22423139 PMC3404712

[4] Sundaram ME, McClure DL, VanWormer JJ, Friedrich TC, Meece JK, Belongia EA. Influenza Vaccination is Not Associated with Detection of Non-Influenza Respiratory Viruses in Seasonal Studies of Influenza Vaccine Effectiveness. Clin Infect Dis. 2013;57:789–93. 23748138 10.1093/cid/cit379 PMC7107973

[5] Wu JT, Ma ES, Lee CK, Chu DK, Ho PL, Shen AL, et al The Infection Attack Rate and Severity of 2009 Pandemic H1N1 Influenza in Hong Kong. Clin Infect Dis. 2010;51:1184–91. 10.1086/656740 20964521 PMC3034199

[6] Chan KH, Peiris JS, Lim W, Nicholls JM, Chiu SS. Comparison of nasopharyngeal flocked swabs and aspirates for rapid diagnosis of respiratory viruses in children. J Clin Virol. 2008;42:65–9. 10.1016/j.jcv.2007.12.003 18242124

[7] Maraun D, Kurths J, Holschneider M. Nonstationary Gaussian processes in wavelet domain: Synthesis, estimation, and significance testing. Physical Review e. 2007;75:016707 17358292 10.1103/PhysRevE.75.016707

[8] Torrence C, Compo GP. A practical guide to wavelet analysis. Bull Amer Meteor Soc. 1998;79:61–78.

[9] Viboud C, Bjornstad ON, Smith DL, Simonsen L, Miller MA, Grenfell BT. Synchrony, waves, and spatial hierarchies in the spread of influenza. Science. 2006;312:447–51. 16574822 10.1126/science.1125237

[10] Yang L, Wong CM, Lau EHY, Chan KP, Ou CQ, Peiris JSM. Synchrony of Clinical and Laboratory Surveillance for Influenza in Hong Kong. PLoS ONE. 2008;3:e1399 10.1371/journal.pone.0001399 18167558 PMC2151138

[11] Yang L, Wong CM, Lau EH, Chan KP, Ou CQ, Peiris JS. Synchrony of clinical and laboratory surveillance for influenza in Hong Kong. PloS ONE. 2008;3:e1399 10.1371/journal.pone.0001399 18167558 PMC2151138

[12] Finkelman BS, Viboud C, Koelle K, Ferrari MJ, Bharti N, Grenfell BT. Global patterns in seasonal activity of influenza A/H3N2, A/H1N1, and B from 1997 to 2005: viral coexistence and latitudinal gradients. PLoS ONE. 2007;2:e1296 18074020 10.1371/journal.pone.0001296 PMC2117904

[13] Maraun D, Kurths J. Cross wavelet analysis: significance testing and pitfalls. Nonlinear Proc Geoph. 2004;11:505–14.

[14] Yang L, Wang XL, Chan KP, Cao PH, Lau HY, Peiris JSM, et al Hospitalisation associated with the 2009 H1N1 pandemic and seasonal influenza in Hong Kong, 2005 to 2010. Euro Surveill. 2012;17:pii = 20309. 23153475

[15] Horby P, Mai le Q, Fox A, Thai PQ, Thi Thu Yen N, Thanh le T, et al The epidemiology of interpandemic and pandemic influenza in Vietnam, 2007–2010: the Ha Nam household cohort study I. Am J Epidemiol. 2012;175:1062–74. 10.1093/aje/kws121 22411862 PMC3353138

[16] Borja-Aburto VH, Chowell G, Viboud C, Simonsen L, Miller MA, Grajales-Muniz C, et al Epidemiological characterization of a fourth wave of pandemic A/H1N1 influenza in Mexico, winter 2011–2012: age shift and severity. Arch Med Res. 2012;43:563–70. 10.1016/j.arcmed.2012.09.005 23079035 PMC3545473

[17] Chuang JH, Huang AS, Huang WT, Liu MT, Chou JH, Chang FY, et al Nationwide surveillance of influenza during the pandemic (2009–10) and post-pandemic (2010–11) periods in Taiwan. PLoS ONE. 2012;7:e36120 10.1371/journal.pone.0036120 . 22545158 PMC3335813

[18] Riley S, Kwok KO, Wu KM, Ning DY, Cowling BJ, Wu JT, et al Epidemiological characteristics of 2009 pandemic influenza based on paired sera from a prospective community cohort. PLoS Med. 2011;8:e1000442 10.1371/journal.pmed.1000442 21713000 PMC3119689

[19] Dierig A, Heron LG, Lambert SB, Yin JK, Leask J, Chow MY, et al Epidemiology of respiratory viral infections in children enrolled in a study of influenza vaccine effectiveness. Influenza Other Respir Viruses. 2014;8:293–301. 10.1111/irv.12229 24483149 PMC4181477

[20] Meningher T, Hindiyeh M, Regev L, Sherbany H, Mendelson E, Mandelboim M. Relationships between A(H1N1)pdm09 influenza infection and infections with other respiratory viruses. Influenza Other Respir Viruses. 2014;8:422–30. 10.1111/irv.12249 24698156 PMC4181801

[21] Yang YW, Wang Z, Ren LL, Wang W, Vernet G, Paranhos-Baccala G, et al Influenza A/H1N1 2009 Pandemic and Respiratory Virus Infections, Beijing, 2009–2010. PLoS ONE. 2012;7:e45807 10.1371/journal.pone.0045807 23029253 PMC3447804

[22] Casalegno JS, Ottmann M, Bouscambert-Duchamp M, Valette M, Morfin F, Lina B. Impact of the 2009 influenza A(H1N1) pandemic wave on the pattern of hibernal respiratory virus epidemics, France, 2009. Euro Surveill. 2010;15:5–7. 20158981

[23] Monto AS. Occurrence of respiratory virus: time, place and person. Pediatr Infect Dis J. 2004;23:S58–S64. 14730271 10.1097/01.inf.0000108193.91607.34

[24] Lau JT, Yang X, Tsui HY, Kim JH. Impacts of SARS on health-seeking behaviors in general population in Hong Kong. Prev Med. 2005;41:454–62. 15917041 10.1016/j.ypmed.2004.11.023 PMC7119319

[25] Chiu SS, Lau YL, Chan KH, Wong WH, Peiris JS. Influenza-related hospitalizations among children in Hong Kong. N Engl J Med. 2002;347:2097–103. 12501221 10.1056/NEJMoa020546

[26] Smith DJ, Lapedes AS, de Jong JC, Bestebroer TM, Rimmelzwaan GF, Osterhaus AD, et al Mapping the antigenic and genetic evolution of influenza virus. Science. 2004;305:371–6. 15218094 10.1126/science.1097211

[27] Centre for Health Protection. Seasonal influenza vaccination coverage survey for the 2012/13 season. Hong Kong: 2013.

[28] USHHS. Healthy People 2020. Available: http://www.healthypeople.gov/2020/.

