# Correction: Impact of the 2009 H1N1 Pandemic on Age-Specific Epidemic Curves of Other Respiratory Viruses: A Comparison of Pre-Pandemic, Pandemic and Post-Pandemic Periods in a Subtropical City

**DOI:** 10.1371/journal.pone.0133946

**Published:** 2015-07-20

**Authors:** Lin Yang, Kwok Hung Chan, Lorna K. P. Suen, King Pan Chan, Xiling Wang, Peihua Cao, Daihai He, J. S. Malik Peiris, Chit Ming Wong

There is information missing from funding section of this paper. The funding section should read: This work was supported by the Health and Medical Research Fund (grants 13121282 and 12111232) and the Area of Excellence Scheme of the University Grants Committee (grant AoE/M-12/-06) of the Hong Kong Special Administrative Region Government. The funders had no role in study design, data collection and analysis, decision to publish, or preparation of the manuscript.
